# Direct measurement of internal temperatures of commercially-available 18650 lithium-ion batteries

**DOI:** 10.1038/s41598-023-41718-w

**Published:** 2023-09-02

**Authors:** Casey M. Jones, Meghana Sudarshan, R. Edwin García, Vikas Tomar

**Affiliations:** 1 School of Aeronautics and Astronautics, Purdue University, West Lafayette, IN 47907 USA; 2 School of Materials Engineering, Purdue University, West Lafayette, IN 47907 USA

**Keywords:** Energy storage, Renewable energy

## Abstract

Direct access to internal temperature readings in lithium-ion batteries provides the opportunity to infer physical information to study the effects of increased heating, degradation, and thermal runaway. In this context, a method to insert temperature sensors into commercial 18650 cells to determine the short- and long-term effects through characterization testing is developed. Results show that sensor insertion only causes a decrease in capacity of 0.5–2.3%, and an increase in DC resistance of approximately 15 mΩ. The temperatures of the modified cells are approximately 0.5 °C higher than the control cells, the difference between the internal and external temperature readings of the modified cells is approximately 0.4 °C, and the modified cells exhibit the same temperature behavior and trend during cycling as the control cells. The cells are able to operate and collect data for 100–150 cycles before their capacities fade and resistances increase beyond what is observed in the control cells. The results of the testing show that cells modified with internal temperature sensors provide useful internal temperature data for cells that have experienced little or no cyclic aging.

## Introduction

The use of lithium-ion batteries (LIBs) has become increasingly common in personal electronics, robotics, grid-independent energy storage, and many other applications^1,2^. The industries for electric vehicles, large-scale power grid and energy storage, and electric aircraft are also growing rapidly in market size and popularity, and are expected to be an integral component in the shift towards clean energy^3–5^. Due to the significant growth in applications and demand, different cell sizes, chemistries, and form factors are continually being tested and improved to further expand the possible applications for LIBs.

One important property to develop new LIB technologies is the internal temperature of a cell during operation. In this context, direct monitoring of the internal temperature during testing provides the opportunity to collect accurate data. When the internal temperature of a cell becomes excessively high, accelerated decomposition of the solid-electrolyte interface (SEI) and breakdown of the electrolyte and separator can occur, which results in thermal runaway that leads to combustion and explosion in the most extreme cases^6,7^. Since internal temperatures during operation are higher than external temperatures, obtaining the internal temperatures of cells can help better understand the thermal response and condition of internal components during testing. Using commercial cells that have been customized with internal temperature sensors can provide more accurate data than modeling internal temperatures based on external characteristics or using custom-designed cells that are not used in commercial applications.

Studies have been conducted to develop methods of monitoring internal temperatures of cells. Li et al. fabricated coin cells with internal temperature sensors to monitor temperatures during external short-circuit testing, which showed a temperature difference of 2–8 °C based on the area of the cathode^8^. Li et al. also fabricated pouch cells with internal temperature sensors to monitor temperatures during overcharge testing, which were able to collect data throughout the thermal runaway and combustion of two cells overcharged at different rates^9^. Hatchard et al. developed a method of inserting a thermocouple into a steel nail to monitor internal cell temperatures during nail penetrations^10^. While these studies provide useful information, they only implement custom-fabricated cells that are not used in commercial applications or can only monitor internal temperatures during physically abusive testing. Directly obtaining internal temperatures of commercially-available cells can provide better indication of possible defects and operational limits during testing in controlled environments, which can provide useful data for the safer design and manufacture of commercial cells.

To monitor the internal temperatures of commercially-available LIBs, it is necessary to develop a method of sensor insertion that will affect cell operation as little as possible. In this paper, a method to insert platinum resistance temperature detectors (RTDs) into commercially-available LIBs is proposed. The impact on the cells is assessed through characterization testing and lifetime cycling and establishes an initial context for the direct correlation between internal temperature measurements and their impact on degradation. Directly obtaining internal temperatures can also be useful to more accurately develop and verify models that estimate internal temperatures using other cell characteristics, and also for directly obtaining internal cell temperatures during physically abusive testing where the behavior of internal temperatures is expected to be appreciably different from external temperatures. The evaluation of the sensor insertion method shows that the modified cells operate similar to unmodified cells for the first 100–150 before indications of accelerated degradation occur. This indicates that data obtained during early cycles could be useful for certain types of tests and provides insight for improvements that can be implemented in the future.

## Results

### Discharge capacities

The operational effects of the RTD insertion on the cells can be seen throughout the lifetime cycle testing. The first indication of the effect on cell performance is the difference between the discharge capacities before and after the RTD insertion is performed on the modified cells. The capacities used for comparison are taken from the final discharge cycle performed at 0.5 C during the characterization testing. Table 1 shows the percent change in discharge capacity after the RTD insertion obtained from the initial cell characterization testing (ICCT).

**Table 1:** Percent decrease in discharge capacity from ICCT cycling after RTD insertion process.

Cell	Percent decrease in discharge capacity (%)
Modified cell 1	2.29
Modified cell 2	0.84
Modified cell 3	1.29
Modified cell 4	0.53

The modified cells show a decrease in discharge capacities in the range of approximately 0.5–2.3%, which could be the result of many different factors. One possible reason is the insertion process likely causes slight physical damage to both the cathode and anode materials, which could also lead to some removal of those materials from the electrodes. Studies have shown that mechanical agitation of cells, such as vibration^11,12^, dynamic impact^13,14^, and partial nail puncture^15,16^, can cause an accelerated loss of discharge capacity beyond what is normally seen during typical cycling. This same effect could be occurring to the cells under test, since the electrodes experience some physical agitation during widening of the center of the windings for RTD insertion. Another possible reason is the evaporation of a small amount of electrolyte occurring while the cells are open to atmosphere during the RTD insertion process. The internal components of the cell are exposed for approximately 2 h in the glovebox during the insertion process, and for a small amount of time afterward while the cells are being resealed. Previous research has shown that common Li-ion battery electrolytes evaporate slowly when exposed to dry ambient air^17^, which would result in only a small decrease in conductivity consistent with the small loss of capacity seen in the modified cells.

**Figure 1:**
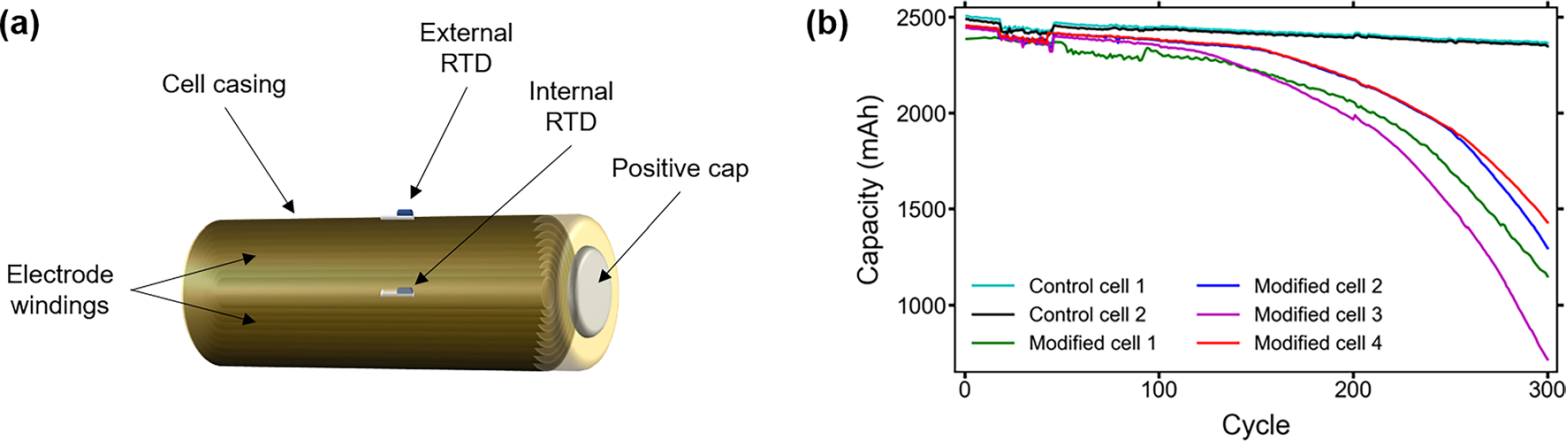
(**a**) Visual representation of cell showing location of internal and external RTDs after insertion procedure; (**b**) discharge capacity of each cell throughout the full testing procedure. The drop in and return to previous capacity seen in the first 100 cycles is due to fluctuating ambient temperatures during testing.

During lifetime cycle testing, the discharge capacities of the modified cells behave similarly to those of the control cells during the first 100–150 cycles. While the RTD insertion causes a small initial difference in discharge capacity between the control and modified cells, this difference is not considerably large until the cells reach approximately halfway through their rated cycle life. Table 2 shows the decrease in discharge capacities for each cell under test after every 100 cycles during the lifetime cycle testing. Figure 1 shows a representation of a cell with the placement of the internal and external RTDs, as well as the discharge capacities of each of the cells throughout lifetime cycle testing.

**Table 2:** Percent decrease in discharge capacity of each cell for every 100 cycles throughout lifetime cycle testing.

Cell	Percent decrease from initial discharge capacity (%)			
	0 cycles	100 cycles	200 cycles	300 cycles
Control cell 1	0	2.18	3.56	5.64
Control cell 2	0	2.15	3.42	5.68
Modified cell 1	0	3.36	14.02	51.80
Modified cell 2	0	3.30	11.59	47.18
Modified cell 3	0	4.11	18.62	70.66
Modified cell 4	0	3.22	11.42	41.78

Approximately halfway through the lifetime cycle testing, the discharge capacities of the modified cells begin decreasing significantly faster than those of the control cells. Capacity loss in LIBs during cycling occurs due to parasitic reactions such as dissolution of transition metals, corrosion of current collectors, electrolyte oxidation at the cathode, and electrolyte reduction at the anode resulting in growth of the SEI layer^18–20^. Extreme cases of parasitic reactions can result in lithium plating and dendrite formation, which can result in micro short circuits that cause accelerated discharge. The accelerated capacity fade that occurs in the modified cells indicates an increased rate of parasitic reactions resulting in reduced output and a shorter useful lifetime^21^. Since the cells are cycled at low C-rates and at ambient temperature, it is expected that the main contribution to the accelerated capacity fade is the loss of electrolyte and growth of the SEI layer. Lithium plating and dendrite formation may provide a small contribution to the accelerated capacity fade, as tests from other researchers have shown evidence they can be present, but it is unlikely that either is a major cause of the accelerated capacity fade. An analysis of the characterization test results and electrochemical impedance spectroscopy (EIS) spectra in the following section further investigates the reasons for the effects on cell discharge capacities.

### Cell temperatures

**Figure 2:**
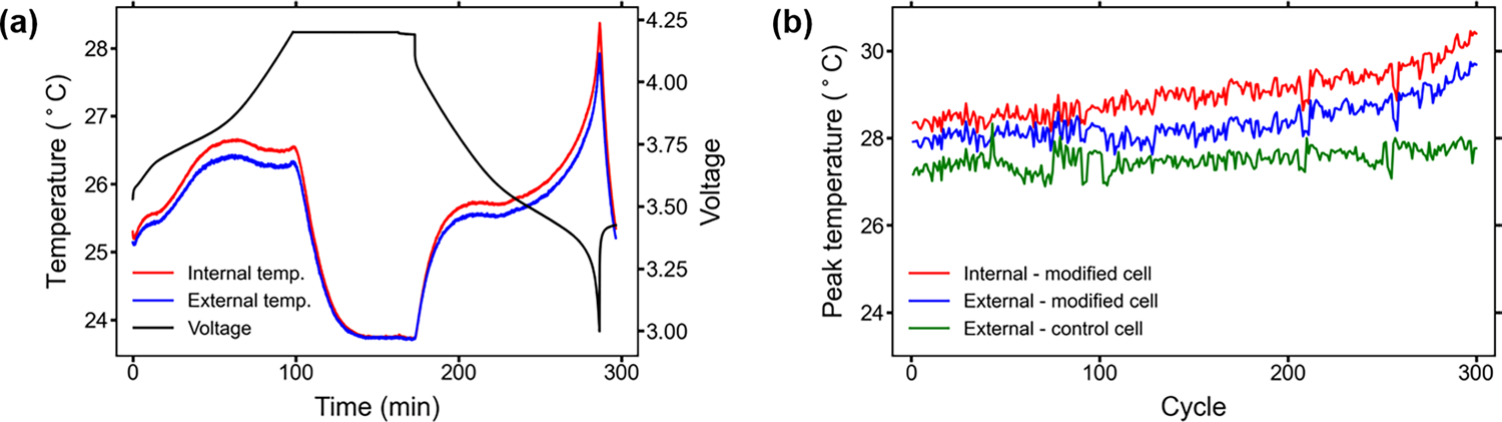
Temperature behavior of cells during testing: (**a**) internal temperature, external temperature, and voltage of a modified cell during a charge and discharge cycle. The temperature peaks at the end of the discharge due to higher resistance causing increased Joule heating; (**b**) peak internal and external temperatures of a modified cell over lifetime compared to peak external temperatures of a control cell. The modified cell peak temperatures increase faster near the end of cell lifetime.

The modified cells show a typical temperature curve throughout the charge and discharge processes. In lithium-ion batteries, heat is generated during charging and discharging through irreversible heat from electrochemical reactions, ohmic (Joule) heating, reversible entropic heat, and contact resistance^22^. Figure 2 shows the temperatures recorded by the internal and external RTDs from the first full charge/discharge cycle of modified cell 1, and the peak temperature per cycle for the internal and external RTDs in a modified cell compared to the peak external temperature of a control cell throughout lifetime cycle testing.

As shown in Fig. 2a, the internal and external temperatures of the modified cell increase during the constant current portion of the charge and reach a maximum difference during charge of approximately 0.3 °C. Both temperatures then decrease to their minimum value during the constant voltage portion of the charge and ensuing rest due to the reduction in ohmic heating and electrochemical reactions from decreasing current. The internal and external temperatures then increase during the discharge, both reaching their peak overall temperatures and also their maximum difference of approximately 0.5 °C at the conclusion of the discharge, when the internal resistance is highest and contribution from Joule heating is most significant^23,24^.

When comparing the peak temperatures during lifetime cycle testing of a modified cell to a control cell as seen in Fig. 2b, the modified cell temperatures during the initial cycles are slightly higher than those of a control cell, with approximately a 0.7 °C difference between external temperatures of the modified and control cells. Throughout approximately the first 100 cycles, the peak temperatures of both the modified and control cells maintain a fairly constant overall level with some noise across the cycles. After this, the control cell exhibits a slight upward trend while the modified cell shows a higher and faster rise in peak temperature throughout the rest of cycling. The final cycles of the testing have the largest difference in temperatures between the modified and control cells, with approximately a 1.8 °C difference between external temperatures. This is the same overall temperature behavior seen in the cells, though results from only one control cell and one modified cell are shown in Fig. 2b for clarity. The suspected reason for the differences in temperature and trend between modified and control cells is the increased internal resistance in the modified cells, which is discussed further in the following section.

## Analysis

### Capacity ratio

**Figure 3:**
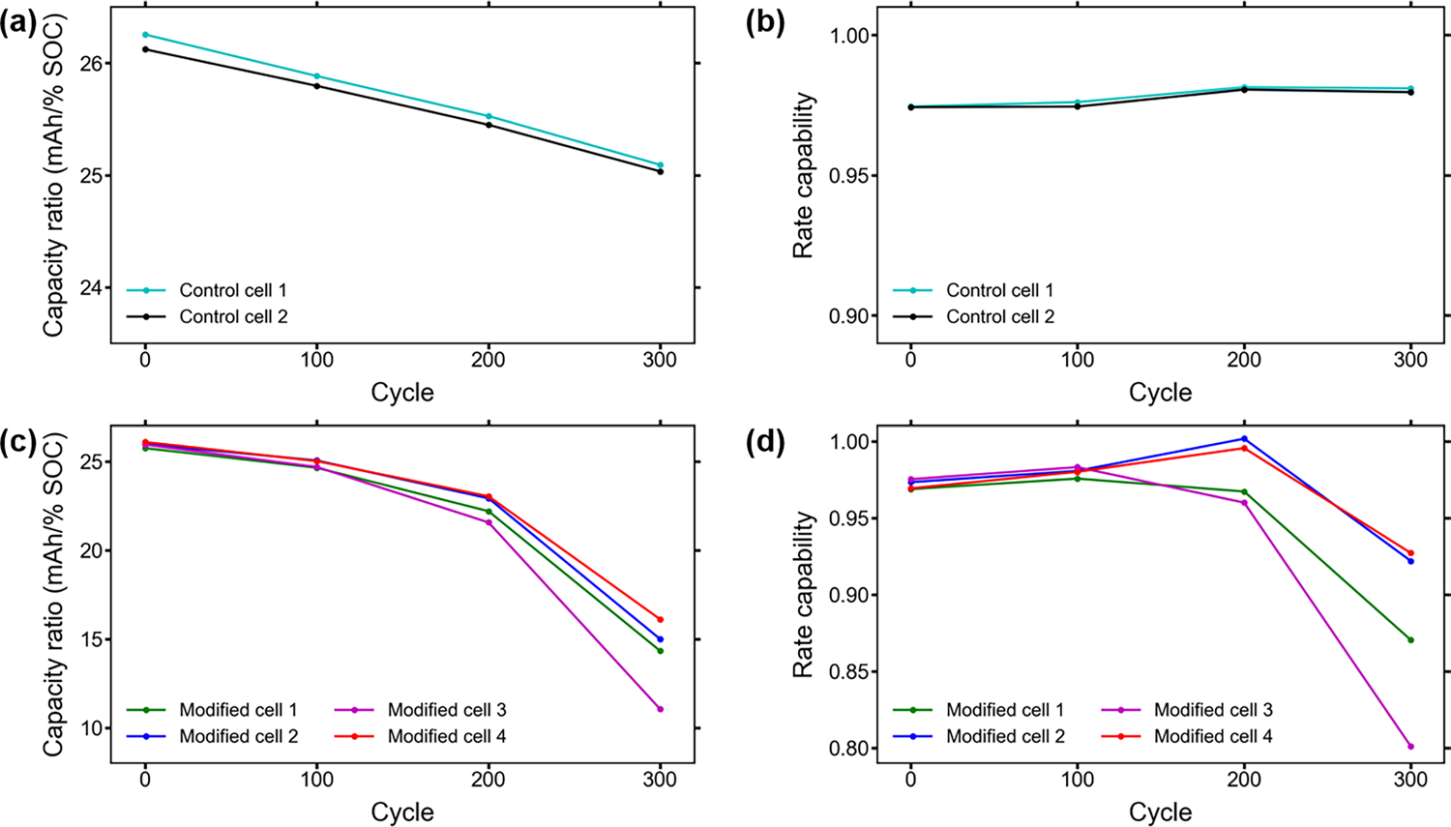
(**a**) Capacity ratio over lifetime for the control cells; (**b**) rate capability over lifetime for the control cells; (**c**) capacity ratio over lifetime for the modified cells. The accelerated decrease in capacity ratio is caused by the decreased availability of active material over lifetime; (**d**) rate capability over lifetime for the modified cells. The discharge capacity at the higher C-rate decreases faster than at the lower C-rate near the end of life, causing the overall decrease in capacity ratio.

Since the capacity ratio of a cell shows how much discharge capacity a cell can provide for a given change in SOC, the capacity ratio over lifetime will behave in the same manner as the capacity fade due to cell degradation from aging. Figure 3a,c show the capacity ratios of the control cells and modified cells calculated from the characterization test data after every 100 aging cycles, which are similar to the discharge capacities of the cells seen in Fig. 1b. The sensor insertion causes a slight decrease in capacity ratio for each of the cells, which continues decreasing as the cells go through more aging cycles.

A decrease in capacity ratio is indicative of a reduction in loading of active material in cells^25^. Therefore, a lower capacity ratio is an indication of less active material being used to provide discharge capacity during cycling. During normal cycling, degradation in cells takes place mostly due to loss of active material (LAM), loss of lithium inventory (LLI), and/or conductivity loss. The severe decrease in capacity ratio seen in the modified cells is due to accelerated LAM, LLI, and conductivity loss caused by the operational effects from the RTD sensor insertion.

LAM is normally caused by structural damage and material loss, whereas LLI is due to SEI growth and lithium plating^26^. The small decrease in capacity ratio seen in the modified cells is due to the LAM that occurs during the physical agitation of the electrodes, inevitable during RTD insertion. Some LAM also occurs during cycling due to the likely higher levels of internal pressure caused by the insertion of the RTD and increased operational temperatures. Higher internal pressure in LIBs caused increased stresses on the electrode materials, especially at a higher state of charge. These stresses result in fatigue cracking of the material that results in capacity fade due to the loss of material and reduced conductivity^27,28^. Lithium plating is not expected to contribute significantly to the accelerated capacity fade, as the cells are cycled at a low C-rate and ambient temperature which does not promote lithium plating^29,30^. SEI growth contributes to impedance in the modified cells, which is discussed further in section "Electrochemical impedance spectroscopy".

### Rate capability

Figure 3b,d show the RTD insertion does not initially have a significant effect on rate capability of the modified cells. In fact, the first 100 cycles show an increase in rate capability of 0.007 to 0.011 for all the modified cells. The next 100 cycles shows an increase in rate capability of 0.015 and 0.021 for two of the modified cells, and a decrease in rate capability of 0.008 and 0.023 for the other two modified cells. The last 100 cycles shows a large decrease in rate capability of 0.068 to 0.159 for all the modified cells. The rate capability of the control cells shows an essentially constant value, with a maximum difference of only around 0.006 between the highest and lowest values. The rate capability of the modified cells shows a maximum difference of 0.07–0.18 between the highest and lowest values, a much larger disparity than the control cells. The decrease in rate capability indicates a higher charge transfer resistance later in life of the modified cells, which causes an increase in resistance and limits the diffusivity of lithium that reduces the discharge capacity at higher C-rates^31,32^.

The large decrease in rate capability during the later stages of aging also indicates a large decrease in conductivity which could be due to increased SEI layer thickness and electrolyte degradation. SEI layer growth causes reduced conductivity between the active materials, causing increased overpotential of a cell that leads to lower rate capability^33,34^. The modified cells that show a larger decrease in rate capability over lifetime also have higher capacity loss, higher DC resistance increase, and higher overall impedance increase.

### DC resistance

**Figure 4:**
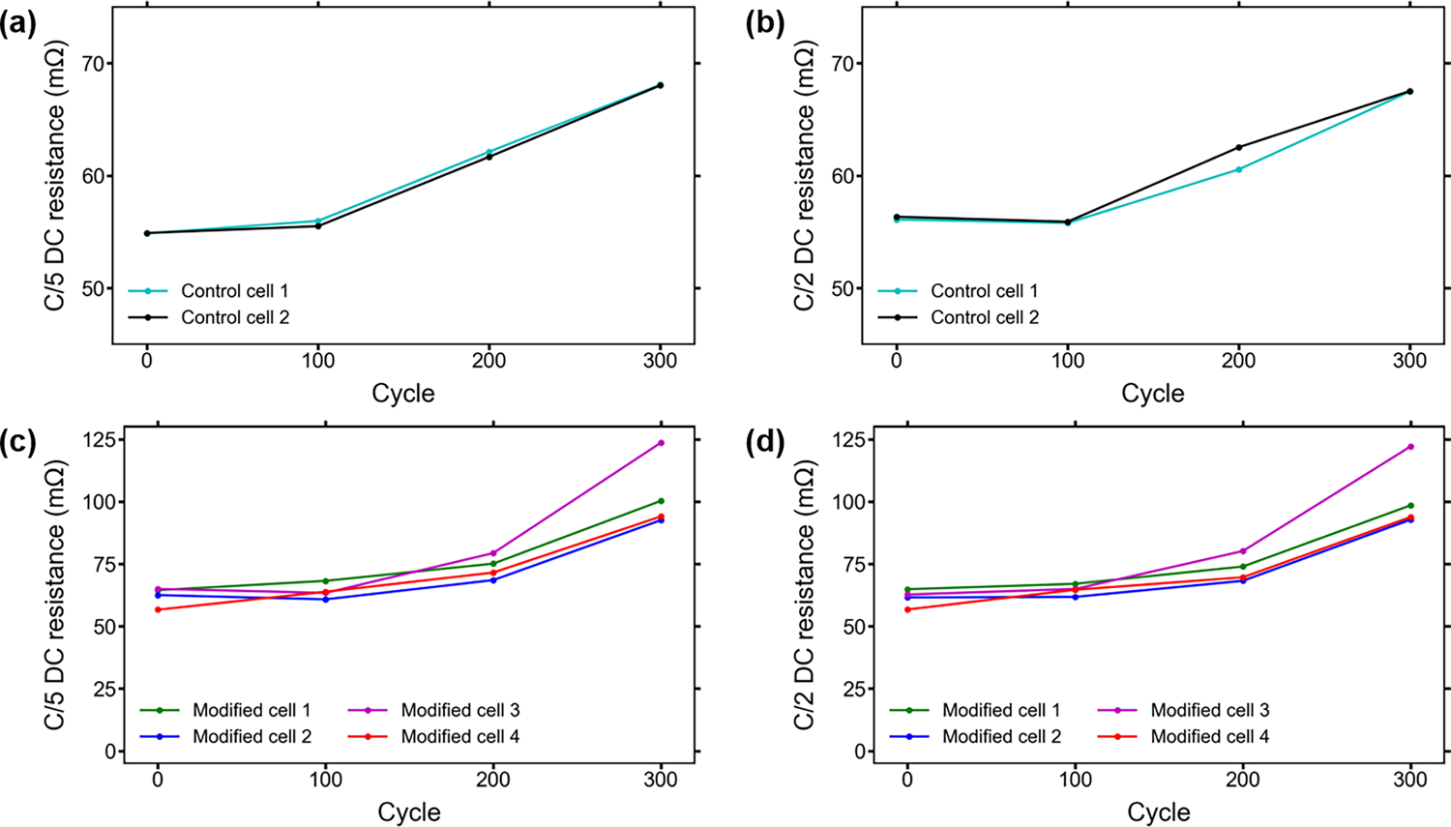
(**a**) DC resistance of control cells calculated using the C/5 discharge from ICCT testing; (**b**) DC resistance of control cells calculated using the C/2 discharge from ICCT testing; (**c**) DC resistance of modified cells calculated using the C/5 discharge from ICCT testing. The higher increase seen in the modified cells is likely due to a loss of conductivity from increased SEI growth and electrolyte loss; (**d**) DC resistance of modified cells calculated using the C/2 discharge from ICCT testing. The C/2 resistance shows essentially the same behavior as the C/5 resistance.

The DC resistance of a cell is dependent on the conductivity of the electrolyte, which is influenced by SOC, ambient temperature, capacity, and other factors^23,35^. Over lifetime the DC resistance of a cell normally increases due to both electrolyte loss from SEI formation and growth, and also electrolyte degradation^36^. Figure 4 shows the DC resistances of the cells after every 100 aging cycles calculated at the beginning of the C/5 and C/2 discharges performed during the characterization tests. There is little noticeable difference between the C/5 and C/2 calculated resistances throughout testing, indicating that both calculations provide essentially the same approximation of DC resistance over lifetime.

The results show that the RTD insertion method causes an increase of approximately 10–15 mΩ in the DC resistance of the modified cells. This is likely due to the evaporation of a small amount of electrolyte while the internal components of the cells are exposed, and could be exacerbated by the physical agitation of the electrodes during the insertion. While the actual resistance values are slightly higher, the DC resistances of the modified cells follow essentially the same behavior as the control cells for the first 200 aging cycles after the RTD insertion procedure. After the aging cycles are completed, the DC resistances of the modified cells exhibit a much larger increase than the control cells.

The most common aging mechanism in LIBs during normal operation is typically the continuous growth of the SEI layer, leading to increased resistance in cells^26^. The drastic increase in resistance seen in the modified cells is an indication that SEI growth could be significantly higher than normal or that less electrolyte is available in the cell, as both would result in conductivity loss. The possible causes of the behavior of the capacity ratio, rate capability, and DC resistance of the cells are further investigated in the following section using the results of the EIS testing.

### Electrochemical impedance spectroscopy

**Figure 5:**
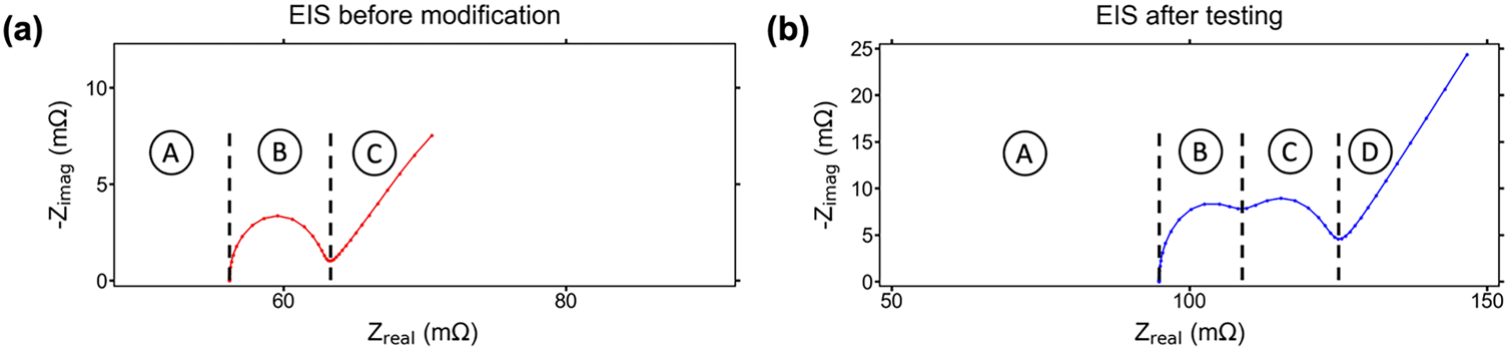
(**a**) EIS spectra before modification. Region A is the Ohmic resistance, region B is the charge transfer resistance, and region C is the Warburg impedance; (**b**) EIS spectra after modification with internal sensors and lifetime cycle testing. Region A is the Ohmic resistance, region B is the SEI impedance, region C is the charge transfer resistance, and region D is the Warburg impedance.

**Figure 6:**
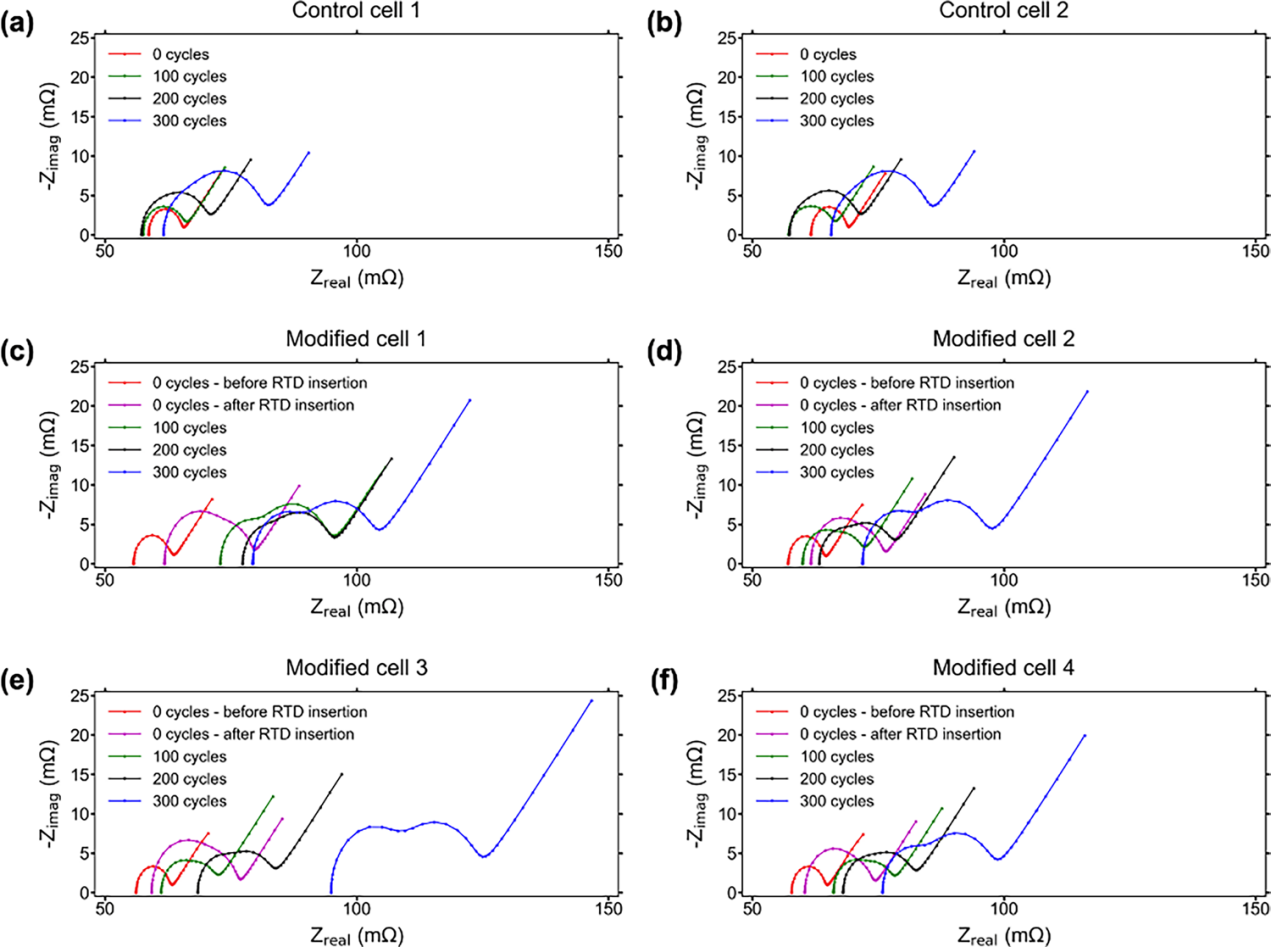
EIS test results for 0 aging cycles, 100 aging cycles, 200 aging cycles, and 300 aging cycles for (**a**) control cell 1; (**b**) control cell 2; (**c**) modified cell 1; (**d**) modified cell 2; (**e**) modified cell 3; and (**f**) modified cell 4. The modified cells show a larger increase in impedance over lifetime, especially at the completion of lifecycle testing.

The results of the EIS testing show the difference in operation between the control cells and modified cells, especially during the later stages of the aging cycling. The first point in the curve at the bottom left, where the EIS spectrum intersects the x-axis, represents the ohmic resistance (R_S_) of a cell and is due to the sum of the resistances of the separator, electrolyte, current collectors, and active material^37^. In general the ohmic resistance of a cell will increase over lifetime, though some variability is seen in the control cells that is normally caused by small local variation when a cell is in the range of 30–80% SOC^38^. Also, during aging, the SEI layer of a cell grows thicker due to depletion of electrolyte and causes an increase in the impedance of the cell. This results in the impedance spectrum shifting further into the capacitive range of impedance, indicated by an upward shift along the y-axis of the overall EIS curve^39^. The impedance behavior of the control cells under test is typical of normal aging where both the capacitive and resistive impedances of the cells show overall increases throughout cycling, indicated by the shift of both cells’ EIS curves up and to the right. Figure 5 shows the different regions of the EIS, and Fig. 6 shows the EIS test results for the two control cells and four modified cells after every 100 cycles throughout testing.

The EIS results from the modified cells show significant differences from the results of the control cells in various ways, especially at the end of the aging cycles. Variations in ohmic resistance, capacitive inductance, spectra shape and behavior, and other differences are seen throughout the lifetime of each of the cells. The first characteristic to point out is the increase in both ohmic and capacitive impedance in the modified cells after the RTD insertion. The reasons are likely that there is a small amount of electrolyte lost from evaporation that causes an increase in ohmic resistance, and the agitation of the electrodes removes a small amount of active material resulting in the increase in capacitive impedance^40^. The possible introduction of oxygen during the RTD insertion process could also be a reason for impedance increase, as the reaction between electrolyte and water vapor causes increased electrolyte degradation resulting in further conductivity loss^41,42^.

Over lifetime the ohmic resistances of the modified cells experience a much larger increase than those of the control cells, similar to the behavior seen in the DC resistances of the cells. The higher initial resistance level of the modified cells, as well as the larger increase in resistance over lifetime, explains the difference in external temperatures between the modified and control cells. A higher resistance causes more Joule heating that results in a higher operational temperature^22^. As explained previously, the modified cells have a slightly higher external temperature at the beginning of the testing, and near the end of testing the temperatures of the modified cells increase at a faster rate. This difference in temperature behavior is due to the higher ohmic resistance that is observed in the modified cells.

The largest visible distinction between the modified and control cells is most clear in the EIS results after the conclusion of aging, where it is seen that the modified cells have two discernible partial semicircles whereas the control cells only have one semicircle. In EIS testing on LIBs, impedance spectra often show one semicircle during early lifetime and a second semicircle appears later in lifetime. A single semicircle represents the impedance contribution from the charge transfer resistance; the appearance and expansion of a second semicircle on the left represents the impedance contribution from the SEI layer^43,44^. This is the reason that at the beginning of testing each of the cells has a single semicircle representing only charge transfer resistance, as the SEI layer is not fully formed and contributes little to cell impedance. Over time the appearance of a second semicircle indicates the full formation and subsequent growth of the SEI layer.

The EIS results indicate that the SEI layer in the modified cells likely experiences significantly more growth than the control cells, causing a much larger increase in impedance over lifetime. Part of the reason for the increased SEI growth in the modified cells could be the operation at slightly higher temperatures over lifetime, as high temperatures reduce SEI stability and lead to increased dissolution causing accelerated growth necessary to replace the SEI^45,46^. These effects may also be caused by the degradation of the electrolyte from the possible exposure to atmosphere. The accelerated growth of the SEI layer also leads to an accelerated depletion of electrolyte, causing further reduction in conductivity and capacity that. This could also be the reason that the modified cells experience a much larger increase in bulk resistance than the control cells, which is signified by a shift along the x-axis of the EIS curves.

The Warburg impedance, which is the diagonally linear portion on the right side of the spectra occurring after the semicircle, reaches a much higher level for the modified cells than the control cells. Warburg impedance is associated with the chemical diffusion process, and is an indication of the diffusion processes occurring in the active material at low frequencies^37,40,47^. The higher Warburg values of the modified cells shows a reduction in the diffusion coefficient, which results in higher impedances of the cells^40,47^. It is difficult to distinguish between the contributions of the different internal components to the rise in overall cell impedance using the results from EIS testing, but the processes typically involved are electrolyte degradation, reduced interfacial conductivity between active material and current collectors, charge transfer processes, and inhibited diffusion, which are also indicated by the results of the characterization tests and lifetime cycling of the cells.

## Methods

### Equipment

The cells used in this study are commercially-available Samsung ICR18650-26J M cylindrical 18650 cells with lithium cobalt oxide (LCO) cathodes and graphite electrodes. The nominal specifications listed in the datasheet^48^ by the manufacturer for the cells are provided in Table 3. The cells used in the testing were disassembled using an MSK-530 disassembling machine for cylindrical cells that utilizes a rotating carbide blade and cylindrical cell-holding die to cut through the cylindrical casing. The internal and external temperature sensors used are Pt100 platinum RTDs with a temperature range of − 50 to ~ 300 °C. The wire used to connect the RTDs is 134-AWP single-conductor polyurethane enamel-coated solid copper wire. The RTD leads and connecting wires are covered with high-temperature lithium battery terminal tape manufactured by MTI. Electrochemical impedance spectroscopy tests are performed using a Potentiostat/Galvanostat/ZRA. The cycling tests are performed using a 5 V/3A 8-channel battery analyzer. Temperature readings from the RTDs are recorded using a data acquisition module.

**Table 3:** Nominal specifications of Samsung 26J 18650 LCO cells^48^.

Nominal capacity (mAh)	Nominal voltage (V)	Charging voltage (V)	Discharge cut-off voltage (V)	Standard charging current (mA)	Cell mass (g)	Cell height (mm)	Cell diameter (mm)	Rated lifetime (cycles)
2600	3.63	4.2 ± 0.05	2.75	1300	45.0	65.0	18.4	~ 300

### Sensor insertion

To obtain operational information on the cells during the experimental process, initial cell characterization tests are performed based on the method developed by researchers at the Hawaii Natural Energy Institute (HNEI)^49^. The procedure developed by HNEI is commonly implemented prior to testing to ensure the cells selected for testing are operating properly and to determine the level of manufacturing variability in a batch of cells. In our work, the tests are implemented before and after the sensor insertion procedure, and multiple times during post-insertion cyclic aging, to observe the immediate and long-term effects that modification has on cell performance. This allows for the stabilization of cell capacity and proper development of the SEI layer prior to sensor insertion; calculation of capacity ratio, rate capability, and DC resistance; observance of impedance increase and capacity decrease; and overall assessment of cell performance. The sensor insertion method, ICCT procedure, and cycling timeline for cell operability testing are outlined in the following paragraphs.

The cells are first cycled six times between the manufacturer-rated charge and discharge cutoff voltages of 4.2 V and 2.75 V, respectively. These cycles are performed at a rate of 0.5 C (1300 mA) to allow for the capacity of the cells to stabilize and establish the initial SEI layer. The cells are then charged to 4.2 V at a rate of 0.5 C and discharged to 2.75 V at a rate of 0.2 C to perform the first cycle of the rate capability test. The cells are then charged and discharged again between the same cutoff voltages, this time at a rate of 0.5 C to perform the second cycle of the rate capability test. The cells are then charged to approximately their nominal voltage of 3.6 V. An EIS test is then performed on each cell to obtain their impedances, and the cells are discharged to 3.0 V to prepare them for sensor insertion.

**Figure 7:**
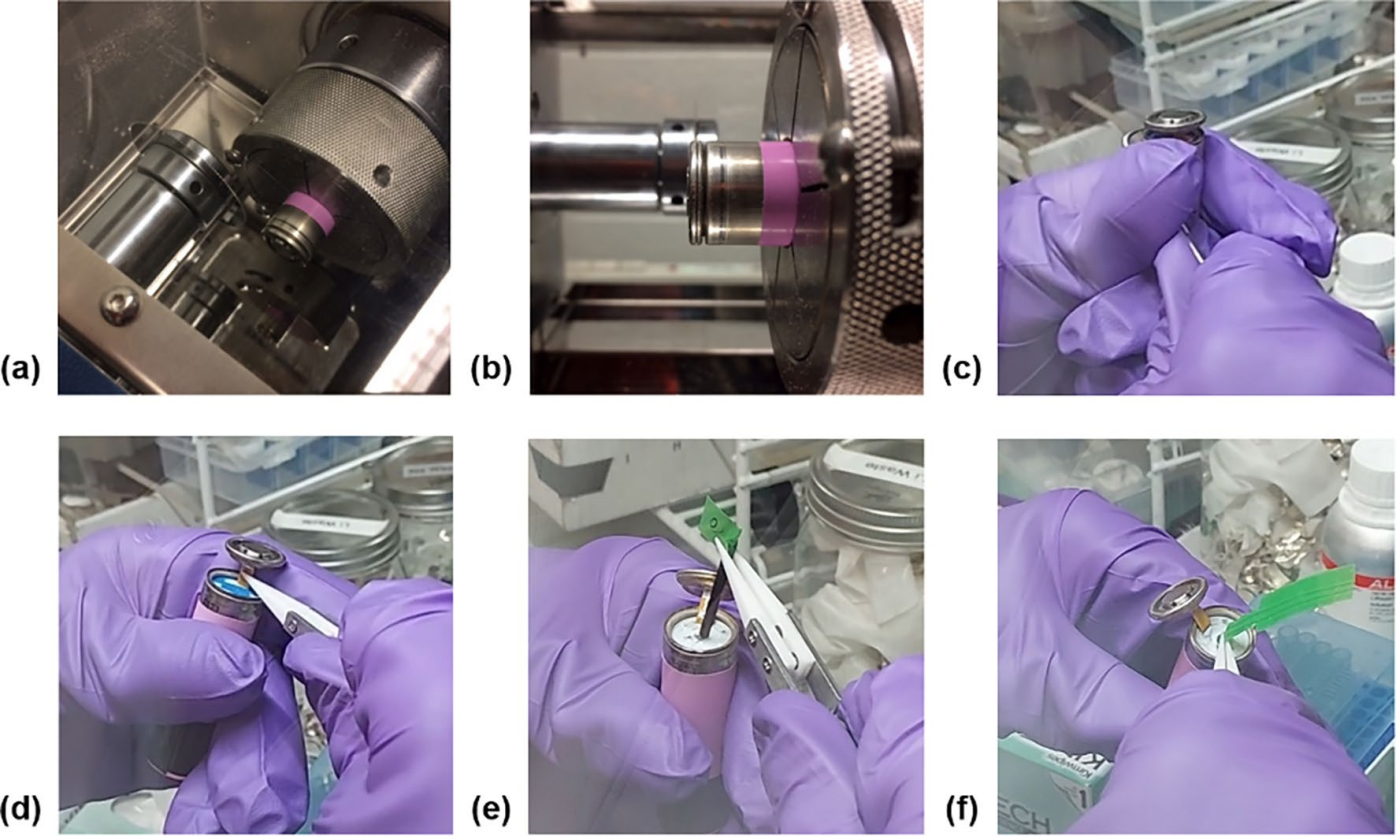
RTD insertion procedure on the commercial 18650 cells: (**a**, **b**) cell being cut by disassembly machine; (**c**–**f**) removal of top of cell, widening of electrode windings, and insertion of RTD into center of cell inside of glovebox.

To begin the RTD insertion method, the cells are individually placed into the disassembling machine and a shallow cut is made around the top circumference of the cell near the positive cap, shown in Fig. 7a,b. The cells are then moved into a glovebox with an inert atmosphere to minimize the amount of oxygen that reaches the internal components. The top of each cell is removed slightly without disturbing the electrical connections, shown in Fig. 7c,d. The opening in the middle of the electrode windings is widened using small rods of increasing diameters, shown in Fig. 7e, and an RTD with leads covered by insulating tape is inserted into the middle of the cell equidistant between the top and bottom edges of the electrodes, shown in Fig. 7f. The top of the cell is replaced and secured with electrical tape, then removed from the glovebox. The area around the top of the cell is then sealed with epoxy to prevent air from entering the cell during operation.

After the RTDs are inserted and the cells are sealed, the ICCTs are partially reperformed to determine the initial effect the RTD insertion process has on cell operation. The six stabilization cycles are no longer necessary as the capacity has already been stabilized, so the cycles with the 0.2 C and 0.5 C discharge are performed to observe the change in cell capacities. The cells are again charged to their nominal voltage and an EIS test is performed to observe the effect on cell impedance. After the modification procedure and characterization tests are complete, the cells are cycled throughout their manufacturer-rated lifetime to determine the effects of the RTD insertion on their long-term operation.

### Characterization testing

The cycling tests implemented for determining long-term effects on cell operation are developed from a NASA testbed used for certifying battery packs in small satellites^50^. Each cell is charged and discharged at a rate of 0.5 C between 3.0 and 4.2 V, including a 10-min rest period after each charge and discharge. After 100 cycles, the partial ICCTs are performed and EIS measurements at nominal voltage are taken. The cells each experience a total of 300 aging cycles, the amount listed by the manufacturer as the rated lifetime. The procedure is carried out on 4 modified cells to observe the variability in results, and on 2 control cells with no modification to provide a performance baseline used to compare operation of the modified cells.

By performing the cell characterization tests before and after the RTD insertion, and also periodically throughout lifetime cycling, the overall methods of cell degradation and reasons for accelerated capacity fade can be investigated. The data gathered from the characterization tests is used to calculate capacity ratio, rate capability, and DC resistance. Capacity ratio is the amount of discharge capacity obtained for each one percent state of charge (SOC) throughout a discharge cycle and is measured in mAh/% SOC. Rate capability is the difference in the ability of a cell to deliver capacity when compared to a baseline discharge and is unitless. A high rate capability is usually desirable as it indicates a cell is capable of providing a more consistent discharge capacity at differing discharge rates. This study uses C/5 (520 mA) as the baseline discharge rate, and C/2 as the comparison discharge rate. Rate capability tests often perform discharges at multiple currents to determine the rate capability over a wide range of different applications, while the characterization tests performed for this work only require one rate capability test for analysis purposes. DC resistance is a measure of the ohmic resistance in a cell due to the contact resistance and conductive resistance (primarily due to the electrolyte) and is measured in mΩ^49^. Equations 1–3 show the calculations for capacity ratio (Qr), rate capability (rC), and DC resistance (R).

1 $${\text{Qr}}=\frac{{{\text{dQ}}}}{{{\text{dSOC}}}} = \frac{{{\text{Q}}_{{\text{C/5}}} }}{{{\text{SOC}}_{{{\text{BOD}}}} - {\text{SOC}}_{{{\text{EOD}}}} }}$$

2 $${\text{rC}}=\frac{{{\text{Q}}_{{\text{C/2}}} }}{{{\text{Q}}_{{\text{C/5}}} }}$$

3 $${\text{R}}=\frac{{{\text{dV}}}}{{{\text{dI}}}}=\frac{{{\text{V}}_{{{\text{BOD}}}} - {\text{V}}_{{{\text{0C}}}} }}{{{\text{I}}_{{{\text{BOD}}}} - {\text{I}}_{{{\text{OC}}}} }}$$

where Q is discharge capacity (for both C/2 and C/5 discharges), SOC_BOD_ is the percent SOC at the beginning of discharge, SOC_EOD_ is the percent SOC at the end of discharge, V_BOD_ is the voltage at the beginning of discharge, V_OC_ is the open circuit voltage at the end of the rest period prior to discharge, I_BOD_ is the current at the beginning of discharge, and I_OC_ is the open circuit current at the end of the rest period prior to discharge (which is zero). The capacity ratio for this study is calculated using the capacity of the C/5 cycle, though the capacity of either the C/5 or C/2 cycle can be used to find capacity ratio without significant difference since the capacity ratio is independent of the cycling rate^51^.

The EIS results are analyzed using the impedance.py package implemented in Python^52^. An adaptive Randles equivalent circuit model (ECM) is used to fit the impedance spectra at low frequencies to analyze the effects on the impedance of the cells throughout testing. An ECM implements different circuit components representing resistance, capacitance, inductance, constant phase elements, and Warburg elements to characterize the impedance behavior of LIBs^53–55^. The ECM uses a resistor element to characterize bulk resistance, a capacitor element in parallel with a resistor element to characterize the SEI arc of the impedance curve, and a capacitor element in parallel with a resistor element and Warburg element to characterize the charge transfer and diffusion arcs of the impedance curve.

## Conclusion

The results of the testing show that the modified cells experience a loss of approximately 0.5–2.3% of their initial capacity due to the sensor insertion procedure. The modified cells show the same capacity fade behavior during early cycles in lifetime testing, then begin to exhibit large changes in operational capabilities around cycle 100–150, limiting their useful lifetime. While the RTD insertion method does affect the operation of the modified cells, these cells are capable of providing useful information on internal temperatures and their relationship to external temperatures in cells that have experienced little or no aging at the time they are used. Future testing of these cells can include physical abuse such as vibration, impact, and nail penetrations, as these tests cause cell temperatures to rise beyond that seen during normal cycling. Further work can also include using internal temperature data to develop and evaluate models that estimate internal temperatures using other cell characteristics.

## Data Availability

The data from the characterization tests may be made available upon reasonable request. Please contact the corresponding author Dr. Vikas Tomar for this purpose.
